# Low Dietary Folate Increases Developmental Delays in the Litters of *Mthfr*^677*TT*^ Mice

**DOI:** 10.3390/nu17152536

**Published:** 2025-08-01

**Authors:** Karen E. Christensen, Marie-Lou Faquette, Vafa Keser, Alaina M. Reagan, Aaron T. Gebert, Teodoro Bottiglieri, Gareth R. Howell, Rima Rozen

**Affiliations:** 1 Departments of Human Genetics and Pediatrics, McGill University, Montreal, QC H3A 0C7, Canada; 2 The Research Institute of the McGill University Health Centre, Montreal, QC H4A 3J1, Canada; 3 The Jackson Laboratory, Bar Harbor, ME 04609, USA; 4 Center of Metabolomics, Institute of Metabolic Disease, Baylor Scott and White Research Institute, Dallas, TX 75204, USA

**Keywords:** MTHFR, methyltetrahydrofolate, homocysteine, folate deficiency, embryo, developmental delay, developmental defects

## Abstract

**Background/Objectives**: Low folate intake before and during pregnancy increases the risk of neural tube defects and other adverse outcomes. Gene variants such as *MTHFR* 677C>T (rs1801133) may increase risks associated with suboptimal folate intake. Our objective was to use BALB/cJ *Mthfr*^677C>T^ mice to evaluate the effects of the TT genotype and low folate diets on embryonic development and MTHFR protein expression in pregnant mice. **Methods**: Female 677CC (mCC) and 677TT (mTT) mice were fed control (2 mg folic acid/kg (2D)), 1 mg folic acid/kg (1D) and 0.3 mg folic acid/kg (0.3D) diets before and during pregnancy. Embryos and maternal tissues were collected at embryonic day 10.5. Embryos were examined for developmental delays and defects. Methyltetrahydrofolate (methylTHF) and total homocysteine (tHcy) were measured in maternal plasma, and MTHFR protein expression was evaluated in maternal liver. **Results**: MethylTHF decreased due to the experimental diets and mTT genotype. tHcy increased due to 0.3D and mTT genotype; mTT 0.3D mice had significantly higher tHcy than the other groups. MTHFR expression was lower in mTT liver than mCC. MTHFR protein expression increased due to low folate diets in mCC mice, whereas in mTT mice, MTHFR expression increased only due to 1D. Developmental delays were increased in the litters of mTT mice fed 1D and 0.3D. **Conclusions**: The *Mthfr*^677C>T^ mouse models the effects of the *MTHFR* 677TT genotype in humans and provides a folate-responsive model for examination of the effects of folate intake and the *MTHFR* 677C>T variant during gestation.

## 1. Introduction

Folate is well-known for its important role in supplying one-carbon units for nucleotide synthesis, methylation reactions, and the detoxification of homocysteine (Hcy) (Figure S1) [1]. One-carbon folate metabolism is particularly important during gestation to support embryonic growth and development [2,3]. Folate supplementation before and during early pregnancy may be required to prevent neural tube defects, heart defects, and other adverse outcomes [4,5,6]. To prevent neural tube defects, it is currently recommended that anyone who may become pregnant supplement with ≥400 μg folic acid/day, and folic acid fortification programs have been implemented in over 70 countries worldwide [1,2,7,8]. In spite of these efforts, suboptimal folate intake in the peri-conceptional period remains common, even in countries with mandatory folic acid fortification [9,10,11,12,13,14,15].

Genetic variants in one-carbon folate metabolism may increase risks associated with sub-optimal folate intake. The *MTHFR* 677C>T gene variant (rs1801133) is a single nucleotide polymorphism that results in the A222V substitution in methylenetetrahydrofolate reductase (MTHFR) [16]. This variant has been identified as a risk factor for neural tube defects, other birth defects and pregnancy complications [17,18,19,20]. The A222V substitution destabilizes the protein and is associated with reduced MTHFR activity, which impairs the reduction of methylenetetrahydrofolate to methyltetrahydrofolate (methylTHF) catalyzed by MTHFR [21]. Homozygosity for the T allele is associated with elevated plasma homocysteine and decreased folates [22,23,24,25]. TT individuals may be more sensitive to low folate intake than CC individuals because the 222VV protein is stabilized by folate [21,26]. In TT individuals, plasma Hcy elevation is greater in those with low folate levels [23,24,27]. Similarly, disease risks associated with the TT genotype may be modified by folate levels [28].

In addition to gene variants, MTHFR activity can be affected by changes in protein expression levels, phosphorylation, and feedback inhibition by S-adenosylmethionine (SAM) [26,29]. MTHFR exists in three different isoforms: unphosphorylated 70 kDa, phosphorylated 70 kDa, and 77 kDa [29,30]. The relative expression of these isoforms is sensitive to dietary folate [26,31]. MTHFR phosphorylation is upregulated when methionine and/or SAM levels are high and increases the sensitivity of the enzyme to SAM inhibition [30,32,33,34,35,36]. These regulatory mechanisms may help to maintain the balance between folate-dependent methylation and nucleotide synthesis [37].

The recently developed *Mthfr*^677C>T^ knock-in mouse model has an alanine-to-valine mutation equivalent to the human A222V variant [38]. This mouse model replicates the reduced MTHFR activity and increased plasma homocysteine associated with the TT genotype, and most importantly, models the thermolability of the variant protein and its stabilization by folates [26,38,39]. The *Mthfr*^677C>T^ mouse is a good model for the investigation of brain and liver abnormalities that are consistent with some human studies on steatosis and Alzheimer’s [26,38,39]. Embryonic development in this mouse model has not yet been examined.

In this study, our objective was to use the *Mthfr*^677C>T^ knock-in mouse to evaluate potential interactions between the TT genotype and low folate diets in embryonic development and MTHFR expression in pregnant mice. We hypothesized that the TT mice would be more sensitive to the effects of folate deficiency than the CC mice.

## 2. Materials and Methods

### 2.1. Mice and Diets

All experiments were performed following Canadian Council on Animal Care guidelines and approved by the Animal Care Committee of the Research Institute of the McGill University Health Centre (protocol 3132). BALB/cJ *Mthfr*^677C>T^ mice were generated by crossing C57BL/6J *Mthfr*^677TT^ (TT) males [38] with BALB/cJ females. Speed congenics were used to select resulting *Mthfr*^677CT^ (CT) males with the highest % BALB/cJ SNPs for successive matings with BALB/cJ females until >90% BALB/cJ background was achieved. BALB/cJ CT females were then mated with BALB/cJ males to ensure the Y chromosome was also BALB/cJ. Two further backcrosses were performed to achieve at least 95% BALB/cJ background. BALB/cJ CT mice were then crossed to obtain the resulting allelic series. Genotyping was performed as in [39]. Mice were group-housed in randomly distributed cages in the same room of a specific pathogen-free facility (18–24 °C, 12 h light-dark cycle, food and water ad libitum). Mice were fed standard chow (Teklad 2918, Inotiv) unless indicated.

Nulliparous *Mthfr*^677CC^ (CC) and TT females were randomly assigned to experimental diets at 4 weeks of age and maintained on that diet until sacrificed. The amino acid-defined experimental diets were supplied by Inotiv (Madison, WI, USA); the formulations are shown in Table S1. The diets have vitamin, mineral, and nutrient contents as recommended for AIN-93G [40,41], with the exception of folic acid, and contain 1% succinylsulfathiazole to inhibit folate synthesis by intestinal bacteria. The control diet (2D), containing 2 mg folic acid/kg diet as in AIN-93G, and folate-deficient diet (0.3D) containing 0.3 mg folic acid/kg diet, were previously described [31]. The 1 mg folic acid/kg diet (1D) differs from 2D and 0.3D only in that the folic acid content was 1 mg folic acid/kg diet, which is twice the minimum amount recommended in the NRC guidelines for mice [42]. The 2 mg folic acid/kg diet was used as the control because this is the most commonly used folic acid content in control diets in mouse studies [43]. Diet consumption was monitored by weighing the food weekly. Average consumption was 2.6 g/mouse/day. Mice were weighed when assigned to diets, after 4 weeks, at mating, and at embryo collection. There were no significant differences in diet consumption or weight gain between the diet/genotype groups (Figure S2).

CC and TT females were mated with CT males after 4–6 weeks on diets (mean: 4.4 weeks; *n*/group: 2D: mCC 23, mTT 17; 1D: mCC 22, mTT 20; 0.3D: mCC 19, mTT 21; CT males: 29; total 151). Embryonic day (E) 0.5 was considered the morning that a vaginal plug was discovered. At E10.5, the females were euthanized by CO_2_ asphyxiation under isoflurane anesthesia. Blood was collected in lithium-heparin tubes via cardiac puncture; embryos, plasma and tissues were collected as described [44]. The developmental stage of the embryos was scored using established morphological markers, as in [44]. Embryos were considered delayed if they were ≥1d behind their most developed littermate. Scoring of embryos for developmental delays and defects was performed by an individual blinded to diet and genotype. Eight sets of twins were observed; twins were excluded a priori from the analysis of delays and defects to avoid confounding effects. Embryos with recoverable tissue were genotyped using DNA extracted from yolk sac, or if necessary, the entire embryo. Embryo sex was determined by PCR as in [45,46]. Embryonic genotype and sex distributions did not differ significantly from expected ratios in any of the diet-maternal genotype groups.

### 2.2. Metabolite Measurement

Plasma 5-methyltetrahydrofolate (MTHF) and total homocysteine (tHcy) were measured by LC-MS/MS as in [47,48].

### 2.3. Western Blots

Protein extracts were prepared as in [26] using RIPA extraction buffer containing Pierce protease inhibitors and phosphatase inhibitors (ThermoFisher, Mississauga, ON, Canada). Immunoblotting was performed as in [26] using primary antibodies specific for methyleneTHF reductase (MTHFR) [16] and ACTIN (A2066, Sigma-Aldrich, Oakville, ON, Canada). Three MTHFR isoforms are detected by immunoblotting: unphosphorylated 70 kDa, phosphorylated 70 kDa, and 77 kDa [29,30]. The 77 kDa isoform is detected just below the phosphorylated 70 kDa band and is very faint relative to the other isoforms in mouse liver. The identity of the phosphorylated and unphosphorylated 70 kDa MTHFR bands has previously been established by phosphatase treatment and mutational analysis [30,49]. Use of ACTIN as a loading control was validated by comparing to normalization by amido black protein staining on the membrane. Data from multiple blots was combined by first normalizing to the 2D group on individual blots (for diet-only blots) or to the 2D mCC group on individual blots (for combined diet and genotype blots).

### 2.4. Statistics

Pregnant female data were analyzed by one- or two-factor ANOVA followed by Tukey post hoc tests. Embryonic genotype and sex distributions were compared using χ^2^ and Fisher’s exact analysis. Categorical data (e.g., defects) were analyzed by binary logistic regression with general linear mixed models (package lme4 [50]) followed by post hoc tests adjusted for multiple testing using the multivariate t method (mvt, package emmeans [51]) using R 4.5.0 [52] in RStudio (version 2025.05.0+496, [53]).

Diet, maternal (m) and embryonic (e) genotypes were specified as fixed effects in the regression models and litter was included as a random effect. Diet and/or genotype interactions were not included in the models because the models without interactions had the lowest Akaike information criterion (AIC) values. To evaluate the effects of embryonic genotype, the litters of mCC and mTT mothers were analyzed separately because mCC litters can have only eCC and eCT embryos and mTT litters can have only eCT and eTT embryos (ie: eCC and eTT cannot be compared). In analyses that included embryonic genotype, the eCT genotype served as the reference. Embryonic sex distributions were not significantly altered by diet, maternal genotype, or embryonic genotype. Sex was not significant and including sex did not improve regression models, so it was not included in the regression analysis. Twins, necrotic, and damaged embryos were excluded a priori from analysis of delays and defects. Individual females and embryos were used as the unit of analysis for calculations. Sample sizes were determined using previous experiments to estimate expected effect sizes and variances [31,44]. Analyses were performed using GraphPad Prism 8.0.1, unless otherwise noted. For all analyses, *p* ≤ 0.05 was considered significant; *p* ≤ 0.08 was considered a trend. *p*-values for all of the variables in the analyses are indicated at the top of the figure panels. Values are presented as mean ± SEM.

## 3. Results

### 3.1. Plasma MethylTHF Decreases and Homocysteine Increases Due to TT Genotype and Low Folate Diet

**Figure 1:**
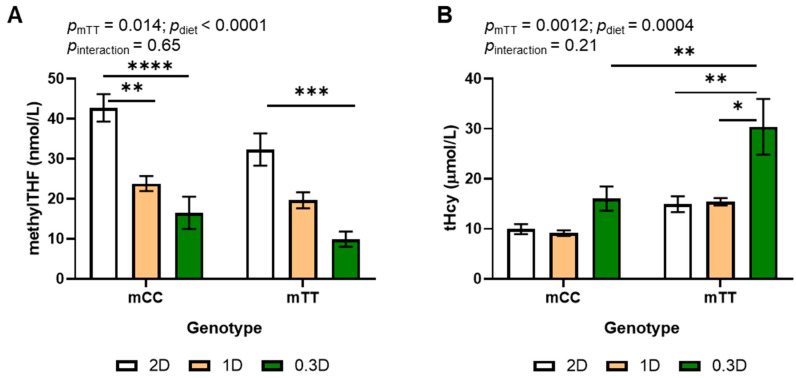
Effects of diet and genotype on (**A**) methylTHF and (**B**) tHcy in maternal plasma of mice fed 2D, 1D, and 0.3D. *n* = 6–9/group, 2-way ANOVA, Tukey post hoc: * *p* < 0.05, ** *p* < 0.005, *** *p* < 0.001, **** *p* < 0.0001.

Decreased folic acid intake due to both the 1D and 0.3D diets significantly decreased plasma methylTHF as compared to 2D (Figure 1A). Plasma methylTHF differed in mice of both genotypes fed the different diets. Plasma methylTHF was significantly lower in 0.3D mice than 2D mice of both genotypes and was significantly lower in mCC 1D mice than mCC 2D mice; there was no difference between 1D and 0.3D in either genotype in post hoc tests. Plasma methylTHF was also significantly lower in mTT (maternal TT) genotype mice as compared to mCC, such that the 0.3D mTT had the lowest concentrations (Figure 1A).

Total plasma homocysteine (tHcy) significantly increased due to both the mTT genotype and the 0.3D diet (Figure 1B). In contrast with methylTHF, 1D had no effect on plasma tHcy concentrations in mice of either genotype as compared to 2D. Plasma tHcy in mTT mice increased approximately 2-fold when fed 0.3D as compared to 1D and 2D, and their tHcy levels were higher than those in mCC at all dietary levels, such that mTT 0.3D mice had significantly higher plasma tHcy than any other group. There was a significant inverse correlation between methylTHF and tHcy across all diets and genotypes (Pearson *r* = −0.5436, *p* < 0.0001, *n* = 48).

### 3.2. Effects of Low Dietary Folate on Hepatic MTHFR Expression Differ Between CC and TT Mothers

**Figure 2:**
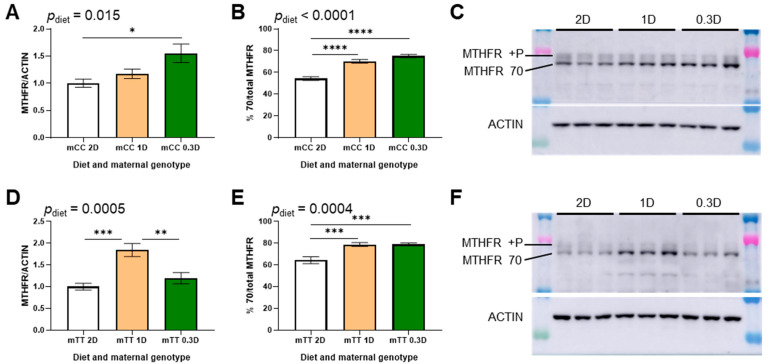
Effects of diet on hepatic MTHFR expression in 2D-, 1D-, and 0.3D-fed mice. (**A**) MTHFR protein expression in CC genotype mothers (mCC; *p*_trend_ = 0.0052). (**B**) MTHFR isoform expression in CC genotype mothers, as % unphosphorylated MTHFR/total (mCC; *p*_trend_ < 0.0001). (**C**) Representative blot of CC genotype mothers (mCC). (**D**) MTHFR protein expression in TT genotype mothers (mTT; *p*_trend_ = 0.28). (**E**) MTHFR isoform expression in TT genotype mothers, as % unphosphorylated MTHFR/total (mTT, *p*_trend_ = 0.0003). (**F**) Representative blot of TT genotype mothers (mTT). *n* = 6/group, 1-way ANOVA, Tukey post hoc: * *p* < 0.05, ** *p* < 0.005, *** *p* < 0.001, **** *p* < 0.0001.

Hepatic MTHFR expression has previously been observed to change in response to folic acid intake [26]. In this study, the effects of the 1D and 0.3D diets on MTHFR protein in liver differed between the mCC and mTT mice. MTHFR protein increased in mCC 0.3D mice as compared to mCC 2D, whereas there was no significant difference in MTHFR protein in mTT 0.3D mice as compared to mTT 2D (Figure 2A,D and Figure S3A). MTHFR protein levels in mCC 1D mice were intermediate between those of mCC 2D and mCC 0.3D mice, and there was a significant trend in the increase in MTHFR protein across the diets in mCC mice (Figure 2A,C). In contrast, in mTT mice, MTHFR protein was significantly higher in 1D mice as compared to both 2D and 0.3D mice (Figure 2D,F). However, MTHFR protein was lower in mTT mice compared to mCC, regardless of diet (Figures S3A and S4A), as expected, due to reduced stability of the mutant protein.

MTHFR phosphorylation was also observed to change due to diet and genotype. In the 1D- and 0.3D-fed mice of both genotypes, the proportion of MTHFR in the unphosphorylated 70 kDa isoform increased significantly due to both low folate diets (Figure 2B,E). The proportion of unphosphorylated 70 kDa MTHFR also increased due to mTT genotype compared to mCC genotype (Figures S3B and S4B).

### 3.3. Low Dietary Folate Increased Developmental Delays in the Litters of TT Mothers

**Figure 3:**
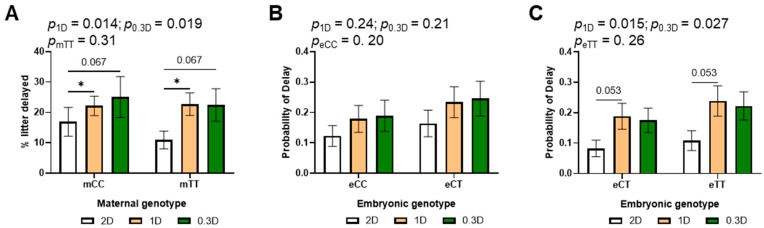
Effects of diet, maternal genotype and embryonic genotype on developmental delays in 2D, 1D, and 0.3D embryos. (**A**) Maternal genotype and diet effects on delay incidence. (**B**) Diet and embryonic genotype effects on probability of delay in litters of mCC mothers. (**C**) Diet and embryonic genotype effects on probability of delay in litters of mTT mothers. (**A**): *n* = 110–159 embryos/group from 17 to 23 litters; (**B**,**C**): *n* = 44–89 embryos/group from 17 to 23 litters; binary logistic regression, mvt post hoc, * *p* < 0.05.

The incidence of developmental delays at E10.5 increased significantly due to both 1D and 0.3D as compared to 2D (Figure 3A, Table S2), although the effects of 1D and 0.3D did not differ significantly from each other. To evaluate the effects of embryonic genotype, the litters of mCC and mTT mothers were analyzed separately because the litters from mCC mothers can have eCC and eCT embryos, while litters from mTT mothers can have eCT and eTT embryos. There was no significant effect of embryonic genotype in the litters of either mCC or mTT mothers (Figure 3B,C). Of interest in this subgroup analysis was the increased likelihood of delays due to 1D and 0.3D, which was significant only in the litters of mTT mothers. This finding is consistent with the diet-genotype effects on tHcy and methylTHF.

**Figure 4:**
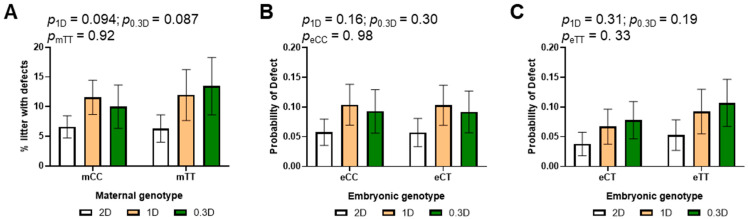
Effects of diet, maternal genotype and embryonic genotype on developmental defects in 2D, 1D, and 0.3D embryos. (**A**) Maternal genotype and diet effects on defect incidence. (**B**) Diet and embryonic genotype effects on probability of defects in litters of mCC mothers. (**C**) Diet and embryonic genotype effects on probability of defects in litters of mTT mothers. (**A**): *n* = 110–159 embryos/group from 17 to 23 litters; (**B**,**C**): *n* = 44–89 embryos/group from 17 to 23 litters; binary logistic regression, mvt post hoc.

Defects observed in the embryos included neural tube defects (open, improper or misaligned neural tube closure), failure to turn, reversed heart looping, lack of development of the telencephalic vesicle, and craniofacial malformations. 80% of embryos with defects were also developmentally delayed. The incidence of defects increased in the litters of 1D- and 0.3D-fed mothers as compared to 2D, but this effect was not significant (Figure 4A, Table S2). As above, there were no significant effects of embryonic genotype in the subgroup analysis of mCC and mTT litters (Figure 4B,C). In addition, there were no significant differences in the likelihood of defects based on maternal genotype.

## 4. Discussion

The *MTHFR* 677C>T gene variant results in a thermolabile form of MTHFR that is associated with decreased MTHFR activity in TT individuals [16,21]. As MTHFR is the only enzyme that catalyzes the reduction of methylenetetrahydrofolate to methylTHF, TT individuals have been shown to have lower serum/plasma and red blood cell folate concentrations than CC individuals [22,23,24,25]. MethylTHF is the one-carbon donor used by methionine synthase to remethylate Hcy, producing methionine. Consequently, decreased MTHFR activity leads to elevated Hcy concentrations in TT individuals compared to CC [22,23,24]. Low folate intake also decreases plasma methylTHF and increases Hcy, and may exacerbate the effects of the TT variant protein by preventing stabilization of the enzyme by folates [23,24,27]. The decreased plasma methylTHF and increased tHcy we observed were expected effects of low folate intake; similarly, the TT genotype effects were expected due to the destabilization of the MTHFR protein by the alanine to valine mutation. In this study, the 0.3D TT mice had the lowest methylTHF and highest Hcy concentrations, which supports the concept that *MTHFR* 677TT individuals are more sensitive to the effects of low dietary folate than CC individuals.

Hcy can also be removed by 2 other metabolic pathways: transsulfuration, which produces cysteine, and folate-independent remethylation via betaine homocysteine methyltransferase, which occur only in specific tissues. In this study, 1D significantly decreased plasma methylTHF as compared to 2D, but Hcy was not increased (Figure 1). In contrast, tHcy was significantly increased by 0.3D. This finding suggests that Hcy remethylation via betaine and/or transsulfuration may compensate for decreased folate intake until a threshold is reached.

MTHFR protein expression has been observed to change in liver in response to folic acid intake [26]. This effect was clearly observed in the mCC mice: MTHFR protein increased significantly across the 2D, 1D and 0.3D diets (Figure 2A), which is consistent with the hypothesis that there is a compensatory mechanism to maintain methylTHF concentrations, presumably for methylation reactions, by increasing MTHFR expression when folate is low. The effect of folic acid intake on MTHFR protein in the mTT mice was less straightforward. As expected, MTHFR protein levels were lower in mTT mice than mCC fed the same diet due to the instability of the variant protein. MTHFR protein in mTT 1D liver was significantly higher than that in mTT 2D liver, consistent with a compensatory mechanism. However, MTHFR protein was significantly lower in mTT 0.3D mice as compared to mTT 1D, and did not differ from the expression in mTT 2D mice. This phenomenon may result from two opposing forces: increased expression in response to low folate, but increased instability of the protein due to a lack of stabilization by folate. That is, increased MTHFR protein may be observed in mTT 1D liver because there is sufficient folate to stabilize the protein, whereas in mTT 0.3D liver there is insufficient folate to stabilize the extra MTHFR, so expression levels stay low.

The phosphorylation of MTHFR is regulated by the concentration of SAM and/or methionine. The unphosphorylated 70 kDa isoform may be more active and/or less sensitive to SAM regulation than the phosphorylated form [30,32,33,34,35]. MTHFR has been reported to be almost completely phosphorylated when methionine and/or SAM levels are high, and conversely, unphosphorylated when Hcy is high [33,36]. These observations are consistent with the increased percentage of unphosphorylated MTHFR observed in 1D- and 0.3D-fed mice of both genotypes. These changes in isoform expression may be a compensatory response to the lower concentrations of methylTHF, which is used as a methyl donor for methionine/SAM synthesis. It is not clear if Hcy has a direct effect on MTHFR phosphorylation. Our observations suggest that Hcy may not be mediating this effect, as MTHFR phosphorylation decreases in 1D mice as compared to 2D mice, while tHcy is unchanged.

This is the first study to investigate developmental anomalies in the recently developed *Mthfr*^677C>T^ mouse model. In this study, we observed increased developmental delays at E10.5 in the litters of mothers fed 1D and 0.3D, which was only significant in the litters of mTT mice (Figure 3). This finding is consistent with the decreased methylTHF and increased tHcy in mTT mice as compared to mCC. It is interesting that increased delays were observed even with 1D, which is not as severe a deficiency as 0.3D. Plasma Hcy is commonly used as marker of folate status, but there was no difference in Hcy in 2D- and 1D-fed mice. This observation suggests that developmental delays may not be caused by toxic effects of Hcy in maternal plasma, and that Hcy concentrations are not a sufficiently sensitive marker of folate deficiencies that affect murine development. At least in mice, there does not need to be a drastic reduction in folate intake to observe changes in methylTHF and developmental delays.

We did not observe any significant effects of embryonic genotype on delay or defects in this study, findings that are consistent with studies on MTHFR knockout mice [54]; however, direct comparisons of eCC, eCT and eTT embryos from CTxCT crosses would be required to rule out embryonic genotype effects. MethylTHF may be actively transported from mother to fetus, as methylTHF in cord blood has been reported to be higher than that in the maternal circulation [55]. MethylTHF is converted to other important folate intermediates [56], but eCT and eTT embryos may obtain enough methylTHF from their mothers at this stage of gestation to compensate for their MTHFR deficiency. Both maternal folate intake and maternal *MTHFR* genotype may influence embryonic growth and development by limiting the overall supply of folate to the embryo that supports nucleotide synthesis and other processes. These observations are consistent with increased risks of growth restriction associated with *MTHFR* 677C>T in some human studies [57]. Developmental defects were increased in the litters of 1D- and 0.3D- fed mothers, but not significantly. Examinations at later time points would be necessary to determine if the incidence of other types of birth defects (e.g., heart defects) is affected in these mice.

Folic acid supplementation has been demonstrated to prevent neural tube defects caused by some mutations or teratogens in certain strains of mice, even though mice may be more resistant to defects caused by folate deficiency than humans [58]. The results of this study are consistent with previous studies showing that folate deficiency alone resulted in developmental delays, but not defects, in wild-type mice [59,60]. It seems that mice may be an imperfect model for developmental defects caused by folate deficiency, such as those associated with the mTT genotype. However, it should be noted that the diets in this study were replete with nutrients other than folate, such as choline and riboflavin. These nutrients may limit the effects of the mTT genotype by enabling compensation for low methylTHF via betaine homocysteine methyltransferase, and stabilization of the TT variant protein by the riboflavin derivative FAD, respectively [21,26]. This may be an important consideration for future studies, as low choline and riboflavin intakes are very common [61,62].

## 5. Conclusions

This BALB/cJ *Mthfr*^677C>T^ mouse model provides a folate-responsive model for the examination of folate intake and the *MTHFR* 677C>T variant during gestation. It could also prove to be useful for studying interactions with other common nutrient deficiencies during development.

## Data Availability

All relevant data are included in this article and Supplemental Materials.

## Supplementary Materials

The following supporting information can be downloaded at: https://www.mdpi.com/article/10.3390/nu17152536/s1, Figure S1: MTHFR and other key enzymes and metabolites in the one-carbon folate pathways; Figure S2: Food consumption, weight gain, body and liver weights in mice fed 2D, 1D and 0.3D; Figure S3: Comparison of maternal genotype groups on hepatic MTHFR expression in 2D- and 0.3D-fed mice.; Figure S4: Comparison of maternal genotype groups on hepatic MTHFR expression in mice fed 1D; Table S1: Formulation of experimental diets; Table S2: Effect of maternal genotype, diet and embryonic genotype on the incidence of delays and defects.

## Abbreviations

The following abbreviations are used in this manuscript:

2D	2 mg folic acid/kg diet
1D	1 mg folic acid/kg diet
0.3D	0.3 mg folic acid/kg diet
CC	*Mthfr*^677CC^
CT	*Mthfr*^677CT^
e	embryonic
E	embryonic day
Hcy	homocysteine
m	maternal
MTHFR	methylenetetrahydrofolate reductase
methylTHF	methyltetrahydrofolate
SAM	S-adenosylmethionine
tHcy	total homocysteine
TT	*Mthfr*^677TT^

## References

[1] Bailey L.B., Stover P.J., McNulty H., Fenech M.F., Gregory J.F., Mills J.L., Pfeiffer C.M., Fazili Z., Zhang M., Ueland P.M. (2015). Biomarkers of Nutrition for Development—Folate Review. J. Nutr..

[2] Crider K.S., Qi Y.P., Yeung L.F., Mai C.T., Head Zauche L., Wang A., Daniels K., Williams J.L. (2022). Folic Acid and the Prevention of Birth Defects: 30 Years of Opportunity and Controversies. Annu. Rev. Nutr..

[3] Graafland N., Rousian M., de Zwart M.L., Steegers-Theunissen R.P.M., Steegers E.A.P., Posthumus A.G. (2025). Parental conditions, modifiable lifestyle factors, and first trimester growth and development: A systematic review. Hum. Reprod. Update.

[4] Zhou Y., Crider K.S., Yeung L.F., Rose C.E., Li Z., Berry R.J., Li S., Moore C.A. (2022). Periconceptional folic acid use prevents both rare and common neural tube defects in China. Birth Defects Res..

[5] Qu Y., Lin S., Zhuang J., Bloom M.S., Smith M., Nie Z., Mai J., Ou Y., Wu Y., Gao X. (2020). First-Trimester Maternal Folic Acid Supplementation Reduced Risks of Severe and Most Congenital Heart Diseases in Offspring: A Large Case-Control Study. J. Am. Heart Assoc..

[6] Yang L., Wang W., Mao B., Qiu J., Guo H., Yi B., He X., Lin X., Lv L., Xu X. (2022). Maternal Folic Acid Supplementation, Dietary Folate Intake, and Low Birth Weight: A Birth Cohort Study. Front. Public Health.

[7] Gomes S., Lopes C., Pinto E. (2016). Folate and folic acid in the periconceptional period: Recommendations from official health organizations in thirty-six countries worldwide and WHO. Public Health Nutr..

[8] Barry M.J., Nicholson W.K., Silverstein M., Chelmow D., Coker T.R., Davis E.M., Donahue K.E., Jaén C.R., Li L., Ogedegbe G. (2023). Folic Acid Supplementation to Prevent Neural Tube Defects: US Preventive Services Task Force Reaffirmation Recommendation Statement. JAMA.

[9] Zhou Y., Wang A., Yeung L.F., Qi Y.P., Pfeiffer C.M., Crider K.S. (2023). Folate and vitamin B12 usual intake and biomarker status by intake source in United States adults aged ≥19 y: NHANES 2007–2018. Am. J. Clin. Nutr..

[10] Cui M., Lu X.L., Lyu Y.Y., Wang F., Xie X.L., Cheng X.Y., Zhang T. (2021). Knowledge and intake of folic acid to prevent neural tube defects among pregnant women in urban China: A cross-sectional study. BMC Pregnancy Childbirth.

[11] Iglesias-Vázquez L., Serrat N., Bedmar C., Pallejà-Millán M., Arija V. (2022). Prenatal folic acid supplementation and folate status in early pregnancy: ECLIPSES study. Br. J. Nutr..

[12] Camier A., Kadawathagedara M., Lioret S., Bois C., Cheminat M., Dufourg M.N., Charles M.A., de Lauzon-Guillain B. (2019). Social Inequalities in Prenatal Folic Acid Supplementation: Results from the ELFE Cohort. Nutrients.

[13] Wegner C., Kancherla V., Lux A., Köhn A., Bretschneider D., Freese K., Heiduk M., Redlich A., Schleef D., Jorch G. (2020). Periconceptional folic acid supplement use among women of reproductive age and its determinants in central rural Germany: Results from a cross sectional study. Birth Defects Res..

[14] Bhide P., Kar A. (2019). Prevalence and determinants of folate deficiency among urban Indian women in the periconception period. Eur. J. Clin. Nutr..

[15] Aweke M.N., Fentie E.A., Agimas M.C., Baffa L.D., Shewarega E.S., Belew A.K., Muhammad E.A., Mengistu B. (2025). Folic acid supplementation during preconception period in sub-Saharan African countries: A systematic review and meta-analysis. PLoS ONE.

[16] Frosst P., Blom H.J., Milos R., Goyette P., Sheppard C.A., Matthews R.G., Boers G.J., den Heijer M., Kluijtmans L.A., van den Heuvel L.P. (1995). A candidate genetic risk factor for vascular disease: A common mutation in methylenetetrahydrofolate reductase. Nat. Genet..

[17] Tabatabaei R.S., Fatahi-Meibodi N., Meibodi B., Javaheri A., Abbasi H., Hadadan A., Bahrami R., Mirjalili S.R., Karimi-Zarchi M., Neamatzadeh H. (2022). Association of Fetal MTHFR C677T Polymorphism with Susceptibility to Neural Tube Defects: A Systematic Review and Update Meta-Analysis. Fetal Pediatr. Pathol..

[18] Yang Y., Chen J., Wang B., Ding C., Liu H. (2015). Association between MTHFR C677T polymorphism and neural tube defect risks: A comprehensive evaluation in three groups of NTD patients, mothers, and fathers. Birth Defects Res. A Clin. Mol. Teratol..

[19] Chen H., Chen X., Yao Q., Xin J., Zhang Y., Huang X., Wang D., Li M., Zhang T., Tillmann T. (2025). Appraising the causal relevance of maternal red blood cell folate and congenital heart disease in offspring: 2-sample Mendelian randomization. Ann. Epidemiol..

[20] Vafapour M., Talebi H., Danaei M., Yeganegi M., Azizi S., Dastgheib S.A., Bahrami R., Pourkazemi M., Jayervand F., Shahbazi A. (2025). Global and population-specific association of MTHFR polymorphisms with preterm birth risk: A consolidated analysis of 44 studies. BMC Pregnancy Childbirth.

[21] Guenther B.D., Sheppard C.A., Tran P., Rozen R., Matthews R.G., Ludwig M.L. (1999). The structure and properties of methylenetetrahydrofolate reductase from Escherichia coli suggest how folate ameliorates human hyperhomocysteinemia. Nat. Struct. Biol..

[22] Fredriksen A., Meyer K., Ueland P.M., Vollset S.E., Grotmol T., Schneede J. (2007). Large-scale population-based metabolic phenotyping of thirteen genetic polymorphisms related to one-carbon metabolism. Hum. Mutat..

[23] Yang Q.H., Botto L.D., Gallagher M., Friedman J.M., Sanders C.L., Koontz D., Nikolova S., Erickson J.D., Steinberg K. (2008). Prevalence and effects of gene-gene and gene-nutrient interactions on serum folate and serum total homocysteine concentrations in the United States: Findings from the third National Health and Nutrition Examination Survey DNA Bank. Am. J. Clin. Nutr..

[24] Colson N.J., Naug H.L., Nikbakht E., Zhang P., McCormack J. (2017). The impact of MTHFR 677 C/T genotypes on folate status markers: A meta-analysis of folic acid intervention studies. Eur. J. Nutr..

[25] Tsang B.L., Devine O.J., Cordero A.M., Marchetta C.M., Mulinare J., Mersereau P., Guo J., Qi Y.P., Berry R.J., Rosenthal J. (2015). Assessing the association between the methylenetetrahydrofolate reductase (MTHFR) 677C>T polymorphism and blood folate concentrations: A systematic review and meta-analysis of trials and observational studies. Am. J. Clin. Nutr..

[26] Christensen K.E., Faquette M.L., Leclerc D., Keser V., Luan Y., Bennett-Firmin J.L., Malysheva O.V., Reagan A.M., Howell G.R., Caudill M.A. (2024). Folic Acid and Methyltetrahydrofolate Supplementation in the Mthfr(677C>T) Mouse Model with Hepatic Steatosis. Nutrients.

[27] Ulvik A., Ueland P.M., Fredriksen A., Meyer K., Vollset S.E., Hoff G., Schneede J. (2007). Functional inference of the methylenetetrahydrofolate reductase 677C > T and 1298A > C polymorphisms from a large-scale epidemiological study. Hum. Genet..

[28] van Beynum I.M., Kapusta L., den Heijer M., Vermeulen S.H., Kouwenberg M., Daniels O., Blom H.J. (2006). Maternal MTHFR 677C>T is a risk factor for congenital heart defects: Effect modification by periconceptional folate supplementation. Eur. Heart J..

[29] Trimmer E.E. (2013). Methylenetetrahydrofolate reductase: Biochemical characterization and medical significance. Curr. Pharm. Des..

[30] Yamada K., Strahler J.R., Andrews P.C., Matthews R.G. (2005). Regulation of human methylenetetrahydrofolate reductase by phosphorylation. Proc. Natl. Acad. Sci. USA.

[31] Christensen K.E., Bahous R.H., Hou W., Deng L., Malysheva O.V., Arning E., Bottiglieri T., Caudill M.A., Jerome-Majewska L.A., Rozen R. (2018). Low Dietary Folate Interacts with MTHFD1 Synthetase Deficiency in Mice, a Model for the R653Q Variant, to Increase Incidence of Developmental Delays and Defects. J. Nutr..

[32] Froese D.S., Kopec J., Rembeza E., Bezerra G.A., Oberholzer A.E., Suormala T., Lutz S., Chalk R., Borkowska O., Baumgartner M.R. (2018). Structural basis for the regulation of human 5,10-methylenetetrahydrofolate reductase by phosphorylation and S-adenosylmethionine inhibition. Nat. Commun..

[33] Zheng Y., Ramsamooj S., Li Q., Johnson J.L., Yaron T.M., Sharra K., Cantley L.C. (2019). Regulation of folate and methionine metabolism by multisite phosphorylation of human methylenetetrahydrofolate reductase. Sci. Rep..

[34] Yamada K., Mendoza J., Koutmos M. (2024). Structural basis of S-adenosylmethionine-dependent allosteric transition from active to inactive states in methylenetetrahydrofolate reductase. Nat. Commun..

[35] Blomgren L.K.M., Huber M., Mackinnon S.R., Bürer C., Baslé A., Yue W.W., Froese D.S., McCorvie T.J. (2024). Dynamic inter-domain transformations mediate the allosteric regulation of human 5, 10-methylenetetrahydrofolate reductase. Nat. Commun..

[36] Büchler L.R., Blomgren L.K.M., Bürer C., Zanotelli V.R.T., Froese D.S. (2025). Evidence for interaction of 5,10-methylenetetrahydrofolate reductase (MTHFR) with methylenetetrahydrofolate dehydrogenase (MTHFD1) and general control nonderepressible 1 (GCN1). Biochimie.

[37] Bhatia M., Thakur J., Suyal S., Oniel R., Chakraborty R., Pradhan S., Sharma M., Sengupta S., Laxman S., Masakapalli S.K. (2020). Allosteric inhibition of MTHFR prevents futile SAM cycling and maintains nucleotide pools in one-carbon metabolism. J. Biol. Chem..

[38] Reagan A.M., Christensen K.E., Graham L.C., Bedwell A.A., Eldridge K., Speedy R., Figueiredo L.L., Persohn S.C., Bottiglieri T., Nho K. (2022). The 677C > T variant in methylenetetrahydrofolate reductase causes morphological and functional cerebrovascular deficits in mice. J. Cereb. Blood Flow. Metab..

[39] Leclerc D., Christensen K.E., Reagan A.M., Keser V., Luan Y., Malysheva O.V., Wasek B., Bottiglieri T., Caudill M.A., Howell G.R. (2024). Folate Deficiency and/or the Genetic Variant Mthfr(677C >T) Can Drive Hepatic Fibrosis or Steatosis in Mice, in a Sex-Specific Manner. Mol. Nutr. Food Res..

[40] Bieri J.G., Stoewsand G.S., Briggs G.M., Phillips R.W., Woodard J.C., Knapka J.J. (1977). Report of the American Institute of Nutrition ad hoc Committee on Standards for Nutritional Studies. J. Nutr..

[41] Reeves P.G. (1997). Components of the AIN-93 diets as improvements in the AIN-76A diet. J. Nutr..

[42] National Research Council (1995). Nutrient Requirements of the Mouse. Nutrient Requirements of Laboratory Animals: Fourth Revised Edition, 1995.

[43] Munezero E., Behan N.A., Diaz S.G., Neumann E.M., MacFarlane A.J. (2022). Poor Reporting Quality in Basic Nutrition Research: A Case Study Based on a Scoping Review of Recent Folate Research in Mouse Models (2009–2021). Adv. Nutr..

[44] Christensen K.E., Malysheva O.V., Carlin S., Matias F., MacFarlane A.J., Jacobs R.L., Caudill M.A., Rozen R. (2021). Mild Choline Deficiency and MTHFD1 Synthetase Deficiency Interact to Increase Incidence of Developmental Delays and Defects in Mice. Nutrients.

[45] McFarlane L., Truong V., Palmer J.S., Wilhelm D. (2013). Novel PCR assay for determining the genetic sex of mice. Sex. Dev..

[46] Tunster S.J. (2017). Genetic sex determination of mice by simplex PCR. Biol. Sex. Differ..

[47] Arning E., Bottiglieri T. (2016). Quantitation of 5-Methyltetrahydrofolate in Cerebrospinal Fluid Using Liquid Chromatography-Electrospray Tandem Mass Spectrometry. Methods Mol. Biol..

[48] Ducros V., Belva-Besnet H., Casetta B., Favier A. (2006). A robust liquid chromatography tandem mass spectrometry method for total plasma homocysteine determination in clinical practice. Clin. Chem. Lab. Med..

[49] Christensen K.E., Mikael L.G., Leung K.Y., Levesque N., Deng L., Wu Q., Malysheva O.V., Best A., Caudill M.A., Greene N.D. (2015). High folic acid consumption leads to pseudo-MTHFR deficiency, altered lipid metabolism, and liver injury in mice. Am. J. Clin. Nutr..

[50] Bates D., Mächler M., Bolker B., Walker S. (2015). Fitting Linear Mixed-Effects Models Using lme4. J. Stat. Softw..

[51] Lenth R.V. (2025). emmeans: Estimated Marginal Means, aka Least-Squares Means.

[52] R Core Team (2025). R: A Language and Environment for Statistical Computing.

[53] RStudio Team (2020). RStudio: Integrated Development for R..

[54] Pickell L., Li D., Brown K., Mikael L.G., Wang X.L., Wu Q., Luo L., Jerome-Majewska L., Rozen R. (2009). Methylenetetrahydrofolate reductase deficiency and low dietary folate increase embryonic delay and placental abnormalities in mice. Birth Defects Res. A Clin. Mol. Teratol..

[55] Kubo Y., Fukuoka H., Kawabata T., Shoji K., Mori C., Sakurai K., Nishikawa M., Ohkubo T., Oshida K., Yanagisawa N. (2020). Distribution of 5-Methyltetrahydrofolate and Folic Acid Levels in Maternal and Cord Blood Serum: Longitudinal Evaluation of Japanese Pregnant Women. Nutrients.

[56] Scott J.M., Weir D.G. (1981). The methyl folate trap. A physiological response in man to prevent methyl group deficiency in kwashiorkor (methionine deficiency) and an explanation for folic-acid induced exacerbation of subacute combined degeneration in pernicious anaemia. Lancet.

[57] Wu H., Zhu P., Geng X., Liu Z., Cui L., Gao Z., Jiang B., Yang L. (2017). Genetic polymorphism of MTHFR C677T with preterm birth and low birth weight susceptibility: A meta-analysis. Arch. Gynecol. Obstet..

[58] Greene N.D., Copp A.J. (2005). Mouse models of neural tube defects: Investigating preventive mechanisms. Am. J. Med. Genet. C Semin. Med. Genet..

[59] Heid M.K., Bills N.D., Hinrichs S.H., Clifford A.J. (1992). Folate deficiency alone does not produce neural tube defects in mice. J. Nutr..

[60] Burgoon J.M., Selhub J., Nadeau M., Sadler T.W. (2002). Investigation of the effects of folate deficiency on embryonic development through the establishment of a folate deficient mouse model. Teratology.

[61] Nguyen H.T., Oktayani P.P.I., Lee S.D., Huang L.C. (2025). Choline in pregnant women: A systematic review and meta-analysis. Nutr. Rev..

[62] McNulty H., Pentieva K., Ward M. (2023). Causes and Clinical Sequelae of Riboflavin Deficiency. Annu. Rev. Nutr..

