# Supplementary material for: Low Dietary Folate Increases Developmental Delays in the Litters of Mthfr677TT Mice
Source: Nutrients. 2025 Aug 1;17(15):2536. doi: 10.3390/nu17152536 (PMC12348234; doi:10.3390/nu17152536)
Supplement: Supplementary file 1 [file nutrients-17-02536-s001.zip › nutrients-3755135-supplementary.pdf]

Table S1: Formulation of experimental diets [1]

Diet Composition (g/kg)	2D (TD.01369) (2 mg/kg folic acid)	1D (TD.240343) (1 mg/kg folic acid)	0.3D (TD.01546) (0.3 mg/kg folic acid)
L-alanine	3.5	3.5	3.5
L-arginine HCl	12.1	12.1	12.1
L-asparagine	6.0	6.0	6.0
L-aspartic acid	3.5	3.5	3.5
L-cystine	3.5	3.5	3.5
L-glutamic acid	40.0	40.0	40.0
Glycine	23.3	23.3	23.3
L-histidine HCl, monohydrate	4.5	4.5	4.5
L-isoleucine	8.2	8.2	8.2
L-leucine	11.1	11.1	11.1
L-lysine HCl	18.0	18.0	18.0
L-methionine	3.3	3.3	3.3
L-phenylalanine	7.5	7.5	7.5
L-proline	3.5	3.5	3.5
L-serine	3.5	3.5	3.5
L-threonine	8.2	8.2	8.2
L-tryptophan	1.8	1.8	1.8
L-tyrosine	5.0	5.0	5.0
L-valine	8.2	8.2	8.2
Sucrose	359.28 [2]	359.18	359.18
Corn starch	150.0	150.0	150.0
Maltodextrin	150.0	150.0	150.0
Soybean oil	80.0	80.0	80.0
Cellulose	30.0	30.0	30.0
Mineral Mix, AIN-93M-MX	35.0	35.0	35.0
Calcium phosphate, monobasic, monohydrate	8.2	8.2	8.2
Succinylsulfathiazole	10.0	10.0	10.0
Niacin	0.03 [3]	0.03	0.03
Calcium pantothenate	0.016 [3]	0.016	0.016
Pyridoxine HCl	0.007 [3]	0.007	0.007
Thiamin HCl	0.006 [3]	0.006	0.006
Riboflavin	0.006 [3]	0.006	0.006
Folic acid	0.002 [3]	0.001	0.0003
Biotin	0.0002 [3]	0.0002	0.0002
Vitamin B_{12} (0.1% in mannitol)	0.025 [3]	0.025	0.025
Vitamin E, d,l-alpha tocopheryl acetate (500 IU/g)	0.15 [3]	0.15	0.15
Vitamin A palmitate (500,000 IU/g)	0.008 [3]	0.008	0.008
Vitamin D_3, cholecalciferol (500,000 IU/g)	0.002 [3]	0.002	0.002
Vitamin K_1, phylloquinone	0.0008 [3]	0.0008	0.0008
Choline bitartrate	2.5	2.5	2.5
Vitamin K, menadione sodium bisulfite	0.05	0.05	0.05
tert-Butylhydroquinone (TBHQ) antioxidant	0.02	0.02	0.02
Green food color	-	-	0.1
Orange food color	-	0.1	-

[1] Diet formulation is based on TD.99366 (a standard amino acid defined diet; Inotiv). Vitamin, mineral and choline content is based on recommendations for AIN-93 (Reeves PG. *J Nutr* 1997; 127:838S-41S); amino acid content is based on Rogers QR and Harper AE. *J Nutr* 1965; 87:267-73. Methionine content is lower than in Rogers and Harper; however, total methionine + cysteine content exceeds the minimum stated in the NRC guidelines (National Research Council. Nutrient Requirements of Laboratory Animals: 4th ed. Washington, DC: National Academies Press (US), 1995).

[2] Sucrose = 349.53 g/kg added sucrose + 9.75 g/kg from Vitamin Mix AIN-93-VX

[3] Obtained from 10 g/kg Vitamin Mix AIN-93-VX

Table S2: Effect of maternal genotype, diet, and embryonic genotype on the incidence of delays and defects

	Maternal Genotype	Diet	n litters	n affected litters	Embryo Genotype	n embryos	n affected embryos
Delay	mCC	2D	23	14	eCC	89	12
					eCT	68	13
		1D	22	17	eCC	70	13
					eCT	80	21
		0.3D	19	14	eCC	44	10
					eCT	63	15
	mTT	2D	17	9	eCT	71	7
					eTT	67	7
		1D	20	15	eCT	71	14
					eTT	65	16
		0.3D	21	15	eCT	75	13
					eTT	72	17
Defects	mCC	2D	23	9	eCC	89	5
					eCT	68	5
		1D	22	11	eCC	70	8
					eCT	80	9
		0.3D	19	7	eCC	44	5
					eCT	63	6
	mTT	2D	17	6	eCT	71	5
					eTT	67	4
		1D	20	9	eCT	71	7
					eTT	65	7
		0.3D	21	12	eCT	75	6
					eTT	72	11

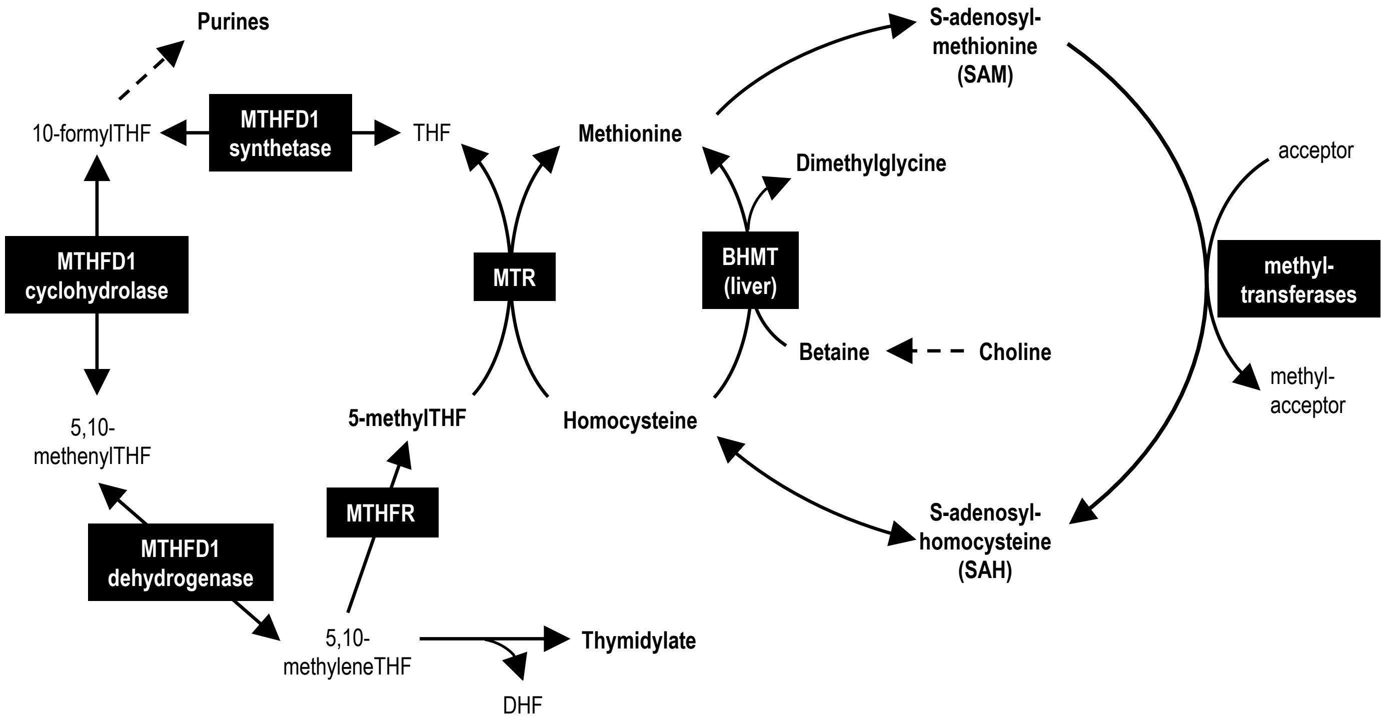

Figure S1: MTHFR and other key enzymes and metabolites in the one-carbon folate pathways. MTHFR produces 5-methylTHF from 5,10-methyleneTHF, committing folate to the methylation cycle. MethylTHF is used by MTR to remethylate homocysteine, producing methionine which can in turn be used to make S-adenosylmethionine, a feedback inhibitor of MTHFR. BHMT is expressed in liver and provides a folate-independent pathway for homocysteine remethylation using betaine as the one-carbon donor.

Abbreviations: BHMT, betaine-homocysteine methyltransferase; DHF, dihydrofolate; DMG, dimethylglycine; MTHFD1, methyleneTHF dehydrogenase-methenylTHF cyclohydrolase-10-formylTHF synthetase; MTR, methionine synthase; MTHFR, methyleneTHF reductase; PEMT, phosphatidylethanolamine N-methyltransferase; SAH, S-adenosylhomocysteine; SAM, S-adenosylmethionine; THF, tetrahydrofolate.

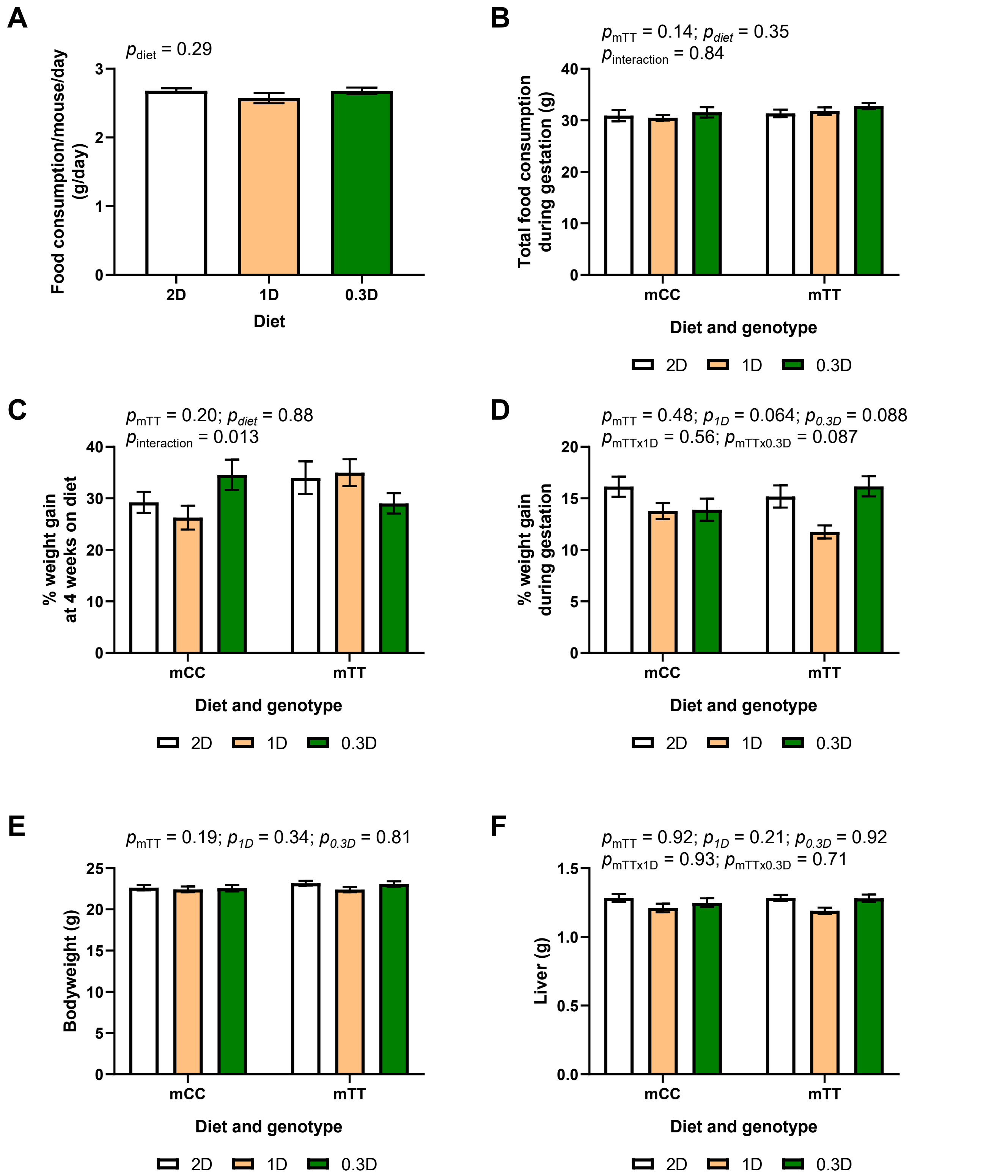

Figure S2. Food consumption, weight gain, body and liver weights in mice fed 2D, 1D and 0.3D. (**A**) Food consumption from t = 0 to 4 weeks on diets. Mice were group-housed prior to mating, so genotype cannot be evaluated. n = 14-17 cages/group, One-way ANOVA, Tukey post-hoc. (**B**) Food consumption during gestation from E0.5 to E10.5. n = 17-23 mice per group, 2-way ANOVA, Tukey post hoc. (**C**) Weight gain t = 0 to 4 weeks on diets, prior to mating. n = 17-22 mice per group, 2-way ANOVA, Tukey post hoc. (**D**) Weight gain during gestation from E0.5 to E10.5. n = 17-22 mice per group, linear mixed-effects regression with diet, genotype, and diet-genotype interaction as fixed effects, and number of decidua as a random effect; mvt post-hoc. (**E**) Maternal body weight at E10.5. n = 17-23 mice per group, linear mixed-effects regression with diet and genotype as fixed effects, and number of decidua as a random effect; mvt post-hoc. (**F**) Maternal liver weight at E10.5. n = 17-23 mice per group, linear mixed-effects regression with diet, genotype, and diet-genotype interaction as fixed effects, and number of decidua and maternal body weight as a random effects; mvt post-hoc. Diet-genotype interactions were included in the regression models shown in panels D-F only if the model with the interaction had the lowest AIC value. There were no significant post-hoc results.

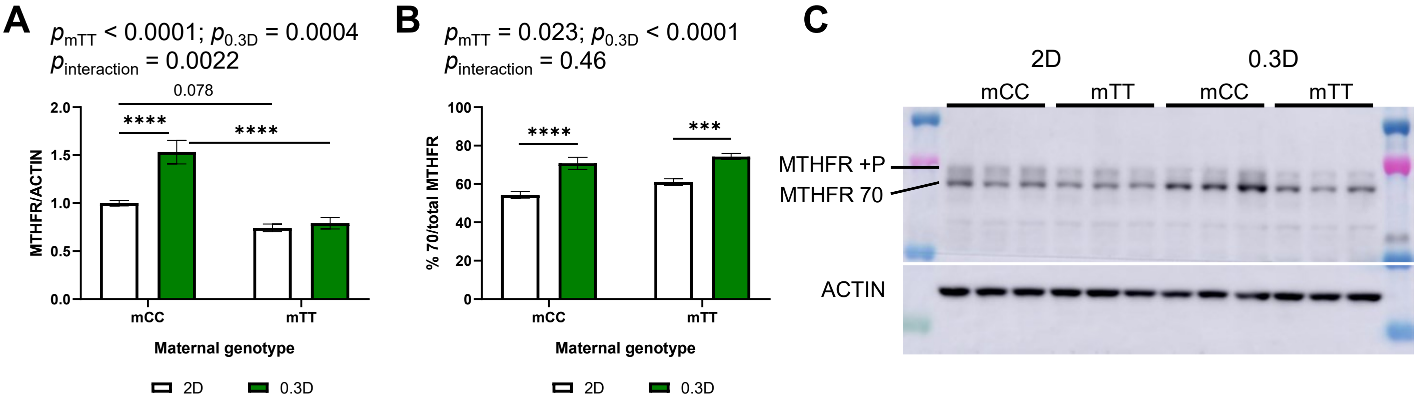

Figure S3. Comparison of maternal genotype groups on hepatic MTHFR expression in 2D- and 0.3D-fed mice. (**A**) MTHFR protein expression. (**B**) MTHFR isoform expression, as % unphosphorylated MTHFR/total. (**C**) Representative blot. n = 9/group, 2-way ANOVA, Tukey post hoc: *** $p < 0.001$ **** $p < 0.0001$.

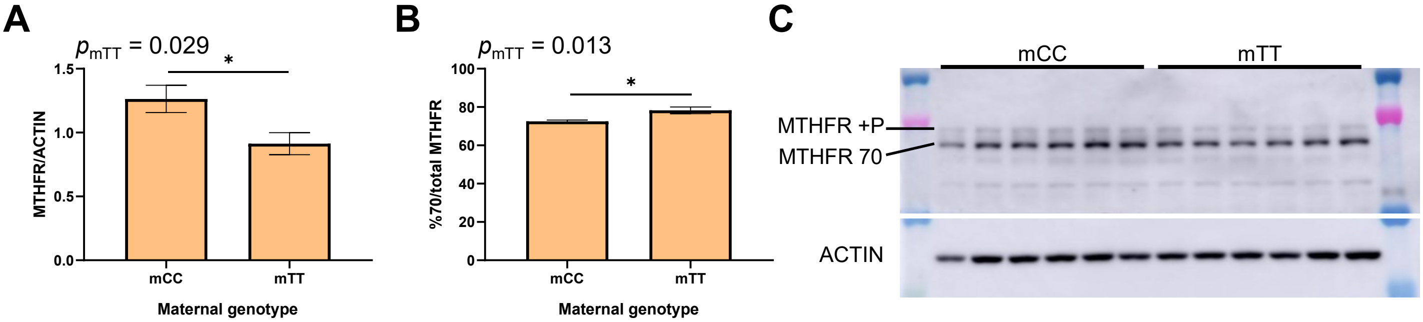

Figure S4. Comparison of maternal genotype groups on hepatic MTHFR expression in mice fed 1D. (**A**) MTHFR protein expression. (**B**) MTHFR isoform expression, as % unphosphorylated MTHFR/total. (**C**) Blot. n = 6/group, *t*-test; * $p < 0.05$.
